# Microenvironmental Signals and Biochemical Information Processing: Cooperative Determinants of Intratumoral Plasticity and Heterogeneity

**DOI:** 10.3389/fcell.2018.00044

**Published:** 2018-04-20

**Authors:** Alexander E. Davies, John G. Albeck

**Affiliations:** ^1^Division of Biological Systems and Engineering, Lawrence Berkeley National Laboratory (LBNL), Berkeley, CA, United States; ^2^Department of Molecular and Cellular Biology, University of California, Davis, Davis, CA, United States

**Keywords:** receptor, kinase, network state transition, neoplastic, epithelial-to-mesenchymal transition, stem cell, single-cell

## Abstract

Intra-tumor cellular heterogeneity is a major challenge in cancer therapy. Tumors are composed of multiple phenotypic subpopulations that vary in their ability to initiate metastatic tumors and in their sensitivity to chemotherapy. In many cases, cells can transition between these subpopulations, not by genetic mutation, but instead through reversible changes in signal transduction or gene expression programs. This plasticity begins at the level of the microenvironment where local autocrine and paracrine signals, exosomes, tumor–stroma interactions, and extracellular matrix (ECM) composition create a signaling landscape that varies over space and time. The integration of this complex array of signals engages signaling pathways that control gene expression. The resulting modulation of gene expression programs causes individual cells to sample a wide array of phenotypic states that support tumor growth, dissemination, and therapeutic resistance. In this review, we discuss how information flows dynamically within the microenvironmental landscape to inform cell state decisions and to create intra-tumoral heterogeneity. We address the role of plasticity in the acquisition of transient and prolonged drug resistant states and discuss how targeted pharmacological modification of the signaling landscape may be able to constrain phenotypic plasticity, leading to improved treatment responses.

## Introduction: two perspectives on tumor heterogeneity

It has long been understood that tumors are composed of multiple cellular subpopulations that vary in their ability to initiate new tumors (Fidler, 1978) and in their sensitivity to chemotherapy (Heppner et al., 1978). When subpopulations of cells within tumors differ markedly in drug resistance, treatment becomes much more difficult, as a single cytotoxic therapy cannot eliminate all of the malignant cells. Heterogeneity in tumor initiation potential also complicates treatment, because even if a therapy kills the vast majority of tumor cells, it will ultimately fail if the few remaining cells are able to expand or disseminate to initiate new tumor sites. Understanding the nature of heterogeneity and the factors that drive it would enable better prediction of effective therapeutic strategies.

Over the past decade, insight into cancer cell heterogeneity has emerged from two distinct fields. First, research into the tumor microenvironment (TME) has revealed that the behavior of tumor cells is dramatically modulated in response to their immediate surroundings; for extensive review see Bissell and Hines (2011) and Quail and Joyce (2013). Mapping of the cellular milieu of tumors in detail has revealed that molecular signals presented by neighboring stromal cells and extracellular matrix (ECM) engage receptors on the surface of tumor cells, triggering intracellular signaling pathways. The resulting induction of gene regulatory circuitry plays a determinative role in the phenotype expressed by a cancer cell. While these signaling pathways are often functionally modified by genetic mutations, DNA sequence alone is insufficient to capture the full range of potential for any cancer cell; mutant cells still respond to extracellular cues, albeit with altered sensitivity. Thus, tumor cell heterogeneity cannot simply be ascribed to genetic diversity within a tumor, but also to the broad variation in signaling cues derived from tumor cells themselves and the many stromal cells that make up the tumor ecosystem (Marusyk et al., 2014; Tabassum and Polyak, 2015).

In parallel, an emerging field has investigated the physical basis of cellular heterogeneity originating in the biochemistry of signaling. From the earliest studies of signal transduction using the *E. coli lac* operon as a model, it has been clear that genetically identical cells respond divergently to environmental stimuli (Novick and Weiner, 1957). At first glance, this variation could be ascribed simply to “noise” in the molecular processes of receptor binding and the relay of intracellular messengers (Korobkova et al., 2004). However, advances in live-cell fluorescence microscopy have made possible well-controlled cell culture experiments that have revealed a deep and intricate underlying structure to the diversity of signaling responses (Levine et al., 2013). Key among these results is the observation that an individual cell's potential to respond to a signaling cue varies from cell to cell and is non-genetic in nature, but is nonetheless heritable for one or more cellular generations (Spencer et al., 2009). Whereas these studies cannot reproduce the physiological complexity of a tumor, they have a clear implication: because the biochemistry of signaling drives variable responses in genetically identical cells even under controlled conditions, the same diversification probably occurs *in vivo* and contributes to the heterogeneity of tumor cells.

The common feature shared by both of these perspectives is the concept that tumor cell heterogeneity can arise from the unique, cell-specific operation of signal transduction pathways within each individual tumor cell. This concept contrasts with the current notion that ongoing genetic mutations are the primary source of heterogeneity in tumors. In reality, both genetic and non-genetic factors contribute substantially to the phenotypic diversity within tumors, but as of yet, there are few approaches that can definitively resolve their relative contributions. The role of intra-tumoral genetic heterogeneity has been reviewed extensively, and for the purposes of this review we defer to other discussions of this topic (Vogelstein et al., 2013; Alizadeh et al., 2015), acknowledging the importance of mutation as a parallel source of phenotypic diversity in tumors. We focus our attention here on how both complex microenvironments and physico-chemical properties of signal transduction cascades contribute to cellular heterogeneity, even in the absence of genetic differences, an important topic that has received more limited attention (Brock et al., 2015).

**Figure 1:**
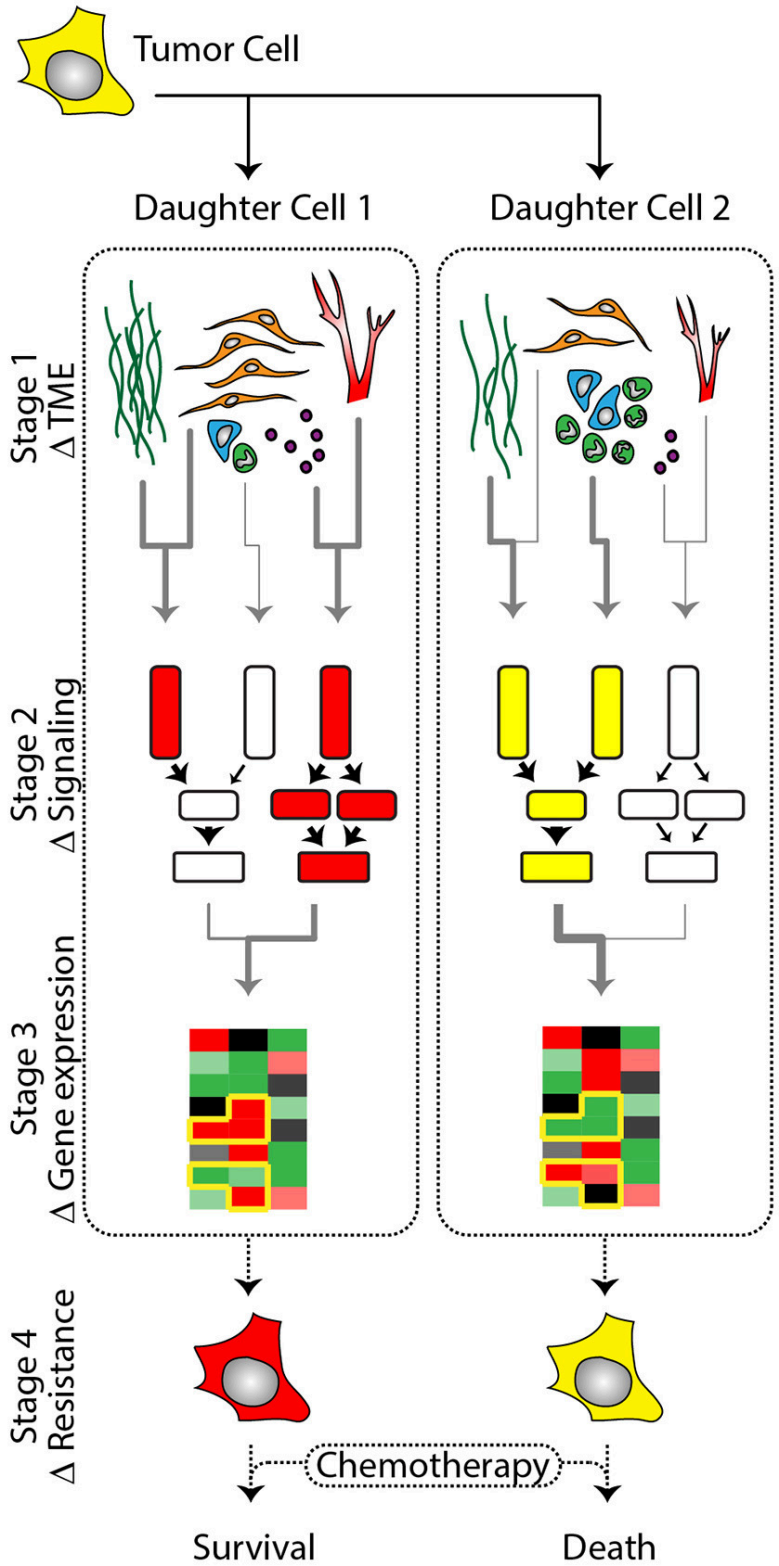
A single tumor cell gives rise to genetically identical daughter cells that vary in phenotype based on exposure to heterogeneous signaling cues and intrinsic variation in signal integration. (Stage 1) Daughter cells are exposed to unique signaling cues in the dynamic tumor microenvironment (TME). Abundance of ECM (dark green), cancer associated- fibroblasts (orange), tumor associated immune cells (blue and green), vasculature (red), and exosomes (purple) vary in abundance and secretory composition throughout the TME, exposing tumor cells to unique signaling microenvironments. (Stage 2) Signals arising from the microenvironment are integrated by membrane receptors and transduced via downstream kinases that modulate transcription factor activation. Inherent cell-to-cell variation in the sensitivity of cells to signaling cues coupled with regional variation in microenvironmental signaling composition contributes to the differential regulation of transcription factors between single cells. (Stage 3) The factors described in Stage 1 and 2 are compounded by transcriptional noise and epigenetic variation leading to cell-to-cell variability in gene expression profiles. (Stage 4) The culmination of microenvironmental signaling and gene expression (Stage 1–3) results in the generation of heterogeneous tumor cell phenotypes (red vs. yellow cells) despite genetically identical backgrounds. As a consequence of phenotypic heterogeneity, some tumor cells will display altered sensitivities when exposed to chemotherapeutic agents, contributing to fractional killing.

As an organizing theme, we present a thought experiment in which two genetically identical tumor cells, originating from the same cell division, experience different microenvironments, and integrate the respective extracellular signals in their gene expression programs, finally resulting in different drug responses (Figure 1). We discuss each stage in this hypothetical divergence, beginning with a discussion of the sources of heterogeneous signals in the microenvironment. We discuss what is understood about variability in the signaling process leading up to regulation of gene expression, followed by the gene expression programs that give rise to persistent phenotypic states and variation in drug resistance. We end with a discussion of how variability in drug sensitivity may be measured and targeted to improve therapeutic responses.

## A landscape of heterogeneous signals

All cells reside in a microenvironment defined by both cellular and non-cellular components. The classic example is the stem cell niche which is a spatially and temporally ordered environment composed of ECM and stromal cells that provide signals to maintain a given cell state (Adams and Watt, 1989; Medema and Vermeulen, 2011; Sato et al., 2011). When a progeny of a stem cell division moves away from the niche, distances of even a few cell widths will expose it to new cues, setting it on a different phenotypic trajectory (e.g., differentiation). The general concept of a niche can be extended to any cell, in the sense that its phenotype is guided by cues from its local microenvironment.

In normal tissues and organs, the microenvironment is organized and maintained over time, supporting homeostasis. However, in cancer the microenvironment becomes corrupted, leading to the formation of many disorganized and heterogeneous niches (Bellail et al., 2004; Lu et al., 2012; Frenkel et al., 2015; Natrajan et al., 2016). As such, the local signals received by an individual tumor cell from fibroblasts, immune lineages, ECM, and/or vascular endothelium vary in composition and strength within the tumor stroma, over small scales. Proximity of tumor cells to stable vs. growing vasculature exposes them to different concentrations of nutrients, hormones, and other cues (Carmeliet and Jain, 2000; Ghajar et al., 2013; Marusyk et al., 2016). The composition of signals secreted by individual stromal cells is also variable due to fibroblast and myeloid subtypes that either co-exist in differing ratios, or are localized in specific regions within the TME (Kiskowski et al., 2011; Carmona-Fontaine et al., 2017). In the following section, we briefly discuss the cell types present in the tumor stroma, examples of their spatial distribution and subtypes, and the signaling cues they provide to shape tumor cell phenotype within the TME. Throughout our descriptions we consider how two daughter cells, resulting from a tumor cell division, could remain in relatively close proximity, yet exhibit heterogeneous phenotypes secondary to the unique sets of extracellular signals within their respective niches (Figure 1, Stage I).

### Cancer-associated fibroblasts (CAF)

Fibroblasts are the most abundant cell type in solid tumors and heavily influence the phenotypic behavior of tumor cells in their local proximity. A compendium of studies have shown that fibroblasts adopt a “cancer-associated fibroblast” (CAF) state, exposing tumor cells in their secretory radius to a host of phenotype-modifying growth factors; for extensive review see (Bhowmick et al., 2004b; Kalluri, 2016). For example, hepatocyte growth factor (HGF), and transforming growth factor β (TGFβ) are known to enhance tumor cell proliferation and promote the acquisition of invasive phenotypes, such as epithelial-to-mesenchymal transitions (EMT) (Stoker et al., 1987; Miettinen et al., 1994; Bhowmick et al., 2004a). Additionally, CAFs produce chemotactic factors and cytokines. Gradients of stromal-derived factor-1 (SDF-1) increase the migratory capacity of tumor cells and provide directionality, whereas cytokines, such as interleukin-6 (IL-6), modulate the proliferation and therapy response characteristics of tumors (Adams et al., 1991; Orimo et al., 2005).

CAFs are not a single entity; they are composed of multiple functionally distinct subtypes and exhibit regional variation in density within the TME (Sugimoto et al., 2006; Kiskowski et al., 2011; Rudnick et al., 2011; Brechbuhl et al., 2017). Early studies hypothesized that CAFs arose from resident fibroblasts that were reprogrammed by tumor-secreted factors. Indeed, it is likely that many CAFs are derived from resident cells. However, recent evidence supports more diverse origins that may contribute to the observed heterogeneity in this population of cells. It has been demonstrated that a proportion of CAFs arise from mesenchymal stems cells (MSC) recruited to the TME from the bone marrow (Worthley et al., 2009). Other mesenchymal cell types, including smooth muscle cells and endothelial cells, have also been implicated as sources of CAF generation (Madar et al., 2013). And, remarkably, tumor cells themselves appear to represent a large CAF reservoir through trans-differentiation from epithelial to mesenchymal states (Zeisberg et al., 2007). These diverse origins raise the possibility that “CAFs” represent not just functional subtypes, but distinct populations of cells that are yet to be defined beyond current morphological and marker expression standards (e.g., smooth muscle actin, vimentin).

As a consequence of their heterogeneity, the signals derived from fibroblast subtypes, and their relative strength in the TME, can alter the phenotypic characteristics of adjacent tumor cells in different ways. For example, in the breast, sub-populations of CD146 or prostaglandin E2 (PG2E) expressing fibroblasts have been identified. These studies show that an increased ratio of CD146(–) fibroblasts suppresses estrogen receptor (ER) expression and renders cells insensitive to tamoxifen (Brechbuhl et al., 2017). However, altering the ratio of CD146(+/−) fibroblasts over time can restore ER expression, and sensitivity to chemotherapy, indicating a non-genetic mechanism by which the stroma can control ER expression in proximal tumor cells. In a similar manner, PGE2(+) fibroblasts enhance tumor growth through secretion of IL-6; thus, controlling the ratio of PGE2(+/−) fibroblast subpopulations can either enhance or suppress tumor growth in a breast cancer model (Rudnick et al., 2011). Finally, CAFs also exhibit focal effects on tumor phenotypes, regulating local growth and invasion characteristics (Gao et al., 2010; Puram et al., 2017). CAFs from the interface zone (i.e., the junction between the tumor and normal tissue) induce a highly invasive tumor phenotype compared to the effect of “normal” fibroblasts or CAFs isolated from the tumor parenchyma (Gao et al., 2010). While the regional and subtype-specific signals produced by these cells are not known, these findings provide compelling evidence for the ability of CAFs to modulate the phenotypic repertoire of tumor cells in a region-specific manner.

### Vasculature, nutrient density, and hypoxia

Vascular networks are of critical importance in tumorigenesis. They deliver oxygen and nutrients vital to sustain rapid growth of tumor cells and provide a conduit for the delivery of immune cells to, and dissemination of tumor cells from, the primary tumor. However, vessels are not passive participants in tumorigenesis; they actively signal to tumor cells to form functional niches (Carmeliet and Jain, 2000). For example, depending upon proximity to a vascular wall, or a growing tip, tumor cell phenotype can be vastly different. Tumor cells residing along a vascular wall niche often take on a cancer stem cell-like (CSC) state and exhibit relative quiescence, or even enter dormant states, based on the local signaling milieu (Calabrese et al., 2007; Ghajar et al., 2013; Malladi et al., 2016). Conversely, cells in niches established by growing vessel tips are exposed to regionally high levels of growth factors, such as TGFβ, and proliferate rapidly (Ghajar et al., 2013). Similarly, vascular integrity is also important. Compromise of the endothelial wall leads to a local influx of platelets and serum proteins into the microenvironment similar to a wound environment. These events are not benign. Platelets release TGFβ and other soluble factors, and serum proteins, such as albumin, contain functional domains that can bind membrane receptors and initiate signal transduction (Laursen et al., 1990; Ivens et al., 2007; Labelle et al., 2011). Nutrient abundance and oxygen tension are also impaired in these regions. The degree of metabolic state change in tumor cells, necrosis, and immune cell infiltration are enhanced by lack of functional vasculature (Helmlinger et al., 1997; Gatenby and Gillies, 2004; Carmona-Fontaine et al., 2017). Thus, signals arising from the vasculature (i.e., perivascular vs. growing tip), or its state of function, are major determinants tumor cell phenotype on a niche-specific basis.

### Immune cells

Immune lineages play important roles in the positive and negative regulation of tumor growth (Wels et al., 2008; Gajewski et al., 2013; Coffelt et al., 2016). Histopathologic characterization of tumors reveal that these cells are functionally and spatially heterogeneous throughout the TME, with the relative abundance of specific immune cell types, or functional sub-states, carrying significant prognostic value (Gooden et al., 2011; Heindl et al., 2015; Natrajan et al., 2016; Tashireva et al., 2017). Due to the complexity of tumoral immunity we will only touch briefly on a few aspects of this important TME component. However, the importance of the immune system in the balance of tumor growth vs. clearance cannot be underscored enough, as is evidenced by the recent surge in immuno-oncology-based therapeutics.

Like CAFs immune cells can take on tumor-associated states. For example, macrophages and neutrophils are reprogrammed to tumor-associated TAM and TAN states, respectively (Fridlender et al., 2009; Egners et al., 2016). Also, similar to CAFs, TAM, and TAN populations can be divided into functional sub-types, referred to as polarization states, which confer either pro- or anti-tumor behaviors to these cells. In the case of TAMs, signals from the TME can polarize their function either toward anti-tumor M1, or tumor promoting M2 states (Chanmee et al., 2014). Neutrophil polarization follows the same respective pattern toward N1 or N2 polarization (Fridlender et al., 2009). Importantly, these states coexist in the TME, with the relative ratios driven by regional signaling inputs.

One TME that favors the polarization of TAMs and TANs into their tumor promoting states is the hypoxic niche (Egners et al., 2016). In this niche, a host of cytokines and growth factors secreted by necrotic and ischemic tumor cells act as potent chemo-attractants (Murdoch and Lewis, 2005). Once recruited to these regions, direct sensing of local oxygen gradients and tumor cell-derived metabolites polarizes TAMs toward the M2 state (Carmona-Fontaine et al., 2017). TGFβ produced by tumor cells perpetuates this transition and also drives the polarization of TANs toward the N2 state (Fridlender et al., 2009). As a result, M2 and N2 cells become coordinate regulators of the local niche structure through production of pro-angiogenic factors, such as VEGF, and secretion of ECM remodeling enzymes. Concurrently, M2 cells release other growth factors, including EGF and TGFβ, that promote tumor proliferation and the migration of tumor cells away from the hypoxic niche. These events generate a microenvironment that promotes tumor expansion and supports metastatic dissemination (Wyckoff et al., 2004; Condeelis and Pollard, 2006; Bonde et al., 2012; Carmona-Fontaine et al., 2017). Therefore, it is not surprising that large necrotic regions and marked infiltration by these cells are clinically correlated with poor prognosis (Vaupel et al., 2001).

T-cells present yet another source of cellular and functional heterogeneity in the TME. Signals released from tumor cells and other immune cells act as potent chemo-attractants for T-cells. However, physical and chemical barriers in the TME impair T-cell localization or function on a region-to-region basis. This is particularly true in the hypoxic niche where signals released from necrotic tumor cells and infiltrating TAMs recruit cytotoxic T-lymphocytes (CTL) to this region (Haddad and Saldanha-Araujo, 2014). However, local derangements in vascular structure often impair infiltration of these tumor-killing cells (Nagy et al., 2009). CTLs that are able to reach this location are inhibited by high levels of TGFβ released from TAMs and tumor cells, and low oxygen tension (Kim et al., 2008, p. 301; Anderson et al., 2017). This leads to impaired cell killing function, shifting the balance in favor of tumor growth within the hypoxic niche. In other regions of the tumor, T-cells are interspersed throughout the parenchyma where the relative ratio of their subtypes determines the net effect on tumor growth. This is particularly true with respect to ratios of CTLs to T regulatory (T_reg_) cells. Like TAMs, T_reg_ cells produce significant amounts of TGFβ, creating a chemical barrier that inhibits CTL proliferation and cell-killing (Anderson et al., 2017). As a result of these actions, increased T_reg_ levels are linked with tumor growth and overall poor prognosis in the clinical population (Gooden et al., 2011).

These vignettes highlight only a limited number of aspects of immune function within tumor tissues, which continue to be investigated intensively. Nonetheless, they begin to establish a mechanism by which different immune components, and variation in their functional states and localization within the TME, carry the potential to modulate tumor cell behavior on a region-to region-basis.

### Extracellular matrix

The ECM provides the structural as well as the signaling foundation regulating normal tissue function. The concept that ECM signals via specific receptors to the nucleus, first proposed by Bissell et al. (1982) has now been demonstrated broadly (Lu et al., 2012). Disruption of ECM composition contributes to the generation of dysfunctional niches, leading to altered cell polarity and ultimately tissue morphology, which are the foundation of dysplastic and neoplastic transitions. We will briefly touch upon a few salient features of the ECM; however, for extensive review we defer to others (Bissell and Hines, 2011; Lu et al., 2012).

Extracellular matrix structure and composition are regulated by multiple cell types in the stroma and affect numerous aspects of tumor cell behavior. Stromal cells, such as fibroblasts, modify the ECM through via production of enzymes which degrade collagens and laminins. Degradation of the ECM releases matrix bound growth factors (e.g., TGFβ, VEGF) and ECM degradation products that stimulate the infiltration of immune cells, promote angiogenesis, and act directly on tumor cells (Bhowmick et al., 2004b). Concurrently, stromal cells produce new fibronectin and laminin forms altering the microenvironment. As such, changes in ECM structure and composition vary with the regional composition of stromal cells within the TME. For example, CAFs, which are enriched at the tumor interface zone, copiously secrete pro-invasive forms of laminin, leading to enrichment of laminin-332 and high levels of TGFβ in this region of the tumor (Kim et al., 2011). Similarly, the interface zone is also enriched with collagen I that is arranged in linear patterns which act as tracks for tumor cells to migrate along (Provenzano et al., 2006; Egeblad et al., 2010). Changes in ECM stiffness and composition are sensed and integrated by tumor cells through integrin-mediated signal transduction pathways that modulate cell polarity and invasive properties (Lühr et al., 2012; Acerbi et al., 2015). Importantly, sensing of ECM stiffness also modulates cellular response characteristics to growth factors, regulating the ability of tumor cells to respond to signaling cues, such as TGFβ, and adopt pro-invasive EMT states (Leight et al., 2012). Thus, extracellular cues arising from matrix composition and stiffness, coupled with its role in direct signaling and signaling modulation (e.g., growth factor storage), make the ECM multifaceted in its ability to control tumor cell fate.

### Emerging signaling mechanisms

Extracellular vesicles have recently gained attention in the cancer field for their roles in intercellular communication; for extensive review see Raposo and Stoorvogel (2013). They are composed of several classes based on size. Microvesicles range in size from 100 to 1,000 nm and are formed by outward budding from the cell membrane (Cocucci et al., 2009). Exosomes range in size from ~30 to 100 nm, are formed via the endocytic system, and are thought to be released by fusion of the multivesicular body with the cell membrane (Ostrowski et al., 2010). Membranous vesicles such as apoptotic bodies also fall under the umbrella of extracellular vesicles and have important biological function; however, we will not discuss them here. Unlike soluble growth factors, extracellular vesicles convey multiple molecular signals in a single packet. They can transport an array of cargo, such as DNA, mRNA, miRNA, and proteins, including functional receptors and multiple growth factors (Thakur et al., 2014; Hoshino et al., 2015; Becker et al., 2016). Importantly, their cargo is highly dependent upon cell type and state. Thus, extracellular vesicle cargos transfer quantitative phenotypic information about host cell state that modify the phenotype of adjacent cells, and importantly, cells in distant microenvironments throughout the body (Peinado et al., 2012; Lázaro-Ibáñez et al., 2017).

The recognized contributions of extracellular vesicles in cancer are growing rapidly. Currently, much of our knowledge comes from studies exploring the role of tumor-derived exosomes on the stroma. Nonetheless these studies provide compelling evidence for the capacity of extracellular vesicles to induce plasticity in their target cells. In the local TME, tumor-derived exosomes and microvesicles activate fibroblasts, myeloid lineages, modulate blood vessel growth and leakiness, and alter ECM structure and composition (Becker et al., 2016). At distant sites such as the lung and liver, tumor-derived exosomes are taken up by resident cells, which leads to phenotypic reprograming and the establishment of premetastatic niches (Peinado et al., 2012; Costa-Silva et al., 2015; Hoshino et al., 2015). Reprogramming of cell state often results in focally altered patterns of ECM deposition, such as increases of fibronectin expression, and regional patterns of immune cell invasion to these areas (Costa-Silva et al., 2015; Hoshino et al., 2015). As a result, circulating tumor cells more readily establish residence at these sites and are able to develop into large metastatic colonies over time.

The role of stromal-derived extracellular vesicles in regulating tumor cell phenotype is less developed; however, the existing data are striking. Current evidence suggests that extracellular vesicles are secreted by all cell types in the TME. CAF-derived exosomes have been shown to promote the sustained growth of tumor cells through transfer of metabolic intermediates, promote breast cancer cell invasion through activation of the cell migration pathways, and modulate therapeutic resistance (Boelens et al., 2014; Zhao et al., 2016; Donnarumma et al., 2017). Exosomes from other stromal sources, such as TAMs, have similarly been shown to modulate the invasive potential of tumor cells (Yang et al., 2011), while endothelial cell exosomes induce vasculogenesis and modulate therapeutic response (van Balkom et al., 2013; Bovy et al., 2015). Finally, microvesicles released from the stroma have been shown to have similar effects compared to exosomes, modulating tumor dormancy vs. growth states and sensitivity to chemotherapeutic agents (Boelens et al., 2014; Sansone et al., 2017a,b). Thus, similar to the effect of soluble signals arising from the stroma, extracellular vesicles appear capable of inducing a wide variety of tumor cell phenotypes.

### Microenvironmental contributions to therapeutic resistance

Several drug tolerant phenotypes have been described in the literature, such as EMT and CSC-like states (Shibue and Weinberg, 2017). Local signals from the TME influence generation of these cell states in a region-specific manner. For example, TGFβ, IL-6, exosomes, and many other CAF-secreted cytokines and growth factors have been shown to drive mesenchymal transitions (Yamada et al., 2013; Boelens et al., 2014; Yu et al., 2014). This results in drug-resistant EMT phenotypes that are enriched in sites like the invasive tumor front where CAFs are densely localized (Nakayama et al., 1998; Puram et al., 2017). Similarly, the hypoxic niche creates a signaling milieu conducive to drug tolerant EMT states (Yang et al., 2008). Finally, regions like the perivascular niche are often enriched with CSCs, which are thought to favor a slow growing phenotype and chemo-resistance (Calabrese et al., 2007; Abdullah and Chow, 2013).

Targeted therapies have attempted to improve the efficacy of treatment by inhibiting specific pathways utilized by, or overexpressed, in cancer. For example, the receptor tyrosine kinase (RTK), epidermal growth factor receptor (EGFR), is frequently mutated in lung cancer, activating the Ras/ERK pathway and driving cell proliferation (Paez et al., 2004). Similarly, another RTK, vascular endothelial growth factor receptor (VEGFR), regulates vessel growth and tumor angiogenesis (Leung et al., 1989). In both cases targeted therapies exist to block the microenvironmental cues that stimulate these pathways (e.g., EGF and VEGF) and their proliferative and pro-angiogenic effects, respectively. However, resistance to these RTK targeted therapies, and even agents that act downstream of these receptors, is frequently observed. In part, this occurs secondary to the complexity of signals arising from stromal components (Junttila and de Sauvage, 2013). CAFs, TAMs, and other cell types in the stroma secrete multiple growth factors, such as EGF, HGF, and PDGF, which activate RTKs through a common Ras/ERK signaling pathway. As such, blockade of EGFR or VEGFR can be overcome by redundant activation of Ras/ERK signaling through other RTKs given the right microenvironmental signaling niche (Straussman et al., 2012; Wilson et al., 2012).

Changes in ECM composition and cell polarity sensed by integrins also drive chemotherapeutic resistance. β4-integrin mediated polarity has been shown to mediate therapy resistance through NF-κB signaling (Weaver et al., 2002). In a similar manner, several studies have shown the fibronectin and other ECM components are capable of inducing resistance through modulation of signaling pathways, such as PI3K/Akt (Pontiggia et al., 2012; Cho et al., 2016). As we have discussed, the ECM is dynamic and variable in composition throughout the TME, implying that resistance-inducing capabilities of the ECM may be variable on a cell-to-cell or regional level.

These brief highlights emphasize the regional variation in stromal composition and signals (cell extrinsic factors) that compose the TME. Returning to our hypothetical daughter cells, we can envision how the two cells, while remaining in relatively close proximity, could sample a variety of extracellular cues as a result of the dynamic and region-specific variability of the TME (Figure 1, Stage 1). In the following section, we will move on to explore how signals are integrated by individual tumor cells, and discuss how intrinsic variation could synergize with variation in signaling from the microenvironment to enhance phenotypic heterogeneity and modulate therapy response.

## Dynamics and diversification in signal transduction

Signal transduction pathways connect extracellular cues to the regulation of gene expression. As our two hypothetical cells receive distinct cues from the components of their TME niche, different intracellular pathways will be stimulated in each one (Figure 1, Stage 2), ultimately leading to different gene expression programs (Figure 1, Stage 3). In principle, this process of stimulated gene expression is profoundly determinative for cell phenotype; it is the orchestrated differential expression of genes that creates the broad diversity of cell types in the adult body. However, at the single-cell level there is inherent variability in signaling and gene expression response to the same signaling cue, blurring the lines between signal input and predictable gene expression output.

### Inherent variability in signaling

Live-cell microscopy with genetically encoded biosensors has revealed that many biochemical events fluctuate continuously within individual cells, with diverse time scales and frequency patterns (Locke and Elowitz, 2009; Levine et al., 2013). Early work in bacteria and yeast explored the physical basis for this variability, and concluded that one potential source is the stochastic nature of biochemical reactions occurring on a very small scale (Elowitz et al., 2002; Volfson et al., 2006). Another source is the heritable propagation of cellular states or properties induced by transient exposure to stimuli unique to each cell. If a subpopulation of cells exposed transiently to a stimulus induces expression of a long-lived gene product, that elevated expression level can persist long after the stimulus is removed, and even through cell division events (Kaufmann et al., 2007). The elevated expression of that gene can then affect the reception and processing of subsequent signals. For example, exposure to a cytokine such as interferon-γ can elevate levels of TNF-receptor, making the cell responsive to TNFα for days after the interferon-γ is removed (Tsujimoto et al., 1986). Studies of cell-to-cell variation have typically categorized these sources as “intrinsic” or “extrinsic,” respectively (Swain et al., 2002).

More recently, studies have investigated the reliability of signaling pathways by quantifying the degree to which information about the concentration of an extracellular stimulus is preserved in the activation of downstream effectors (Cheong et al., 2011). Surprisingly, most signaling pathways have a measured channel capacity of only 1–2 bits, meaning they can respond differentially to, at most, 2–4 different concentrations of the extracellular stimulus (Uda et al., 2013; Selimkhanov et al., 2014). More work remains to be done to refine these measurements, but the emerging view is that the intracellular response to a given signal is far from an absolute and precise measurement of the extracellular cue; instead, it is contingent on the pre-existing state of the cell, the biochemical limits of the pathway (for example, saturation of a particular step in the pathway), and thermodynamically stochastic events.

### Variation over time: dynamic heterogeneity in signal transduction

If we were able to directly monitor multiple signaling pathways within our two hypothetical cells, we would likely observe continual fluctuations and pulses of activity as they respond to both static and evolving cues in their microenvironment. Cancer-relevant signaling pathways, such as p53, Ras/ERK, and NF-kB, respond to constant stimuli in the form of discrete pulses that ultimately influence gene expression and ultimately cell phenotype; however, the characteristics of these responses vary on the cell-to-cell level.

Lahav and colleagues demonstrated that the transcription factor p53 is activated in discrete hour-long pulses following a DNA-damaging event (Lahav et al., 2004). Further investigation revealed significant heterogeneity in single-cell p53 responses to fixed concentrations of cisplatin. Under these conditions, cells that accumulated p53 at a rapid rate (1–2 days) underwent apoptosis, whereas cells that accumulated the same peak levels of p53 over several days survived (Paek et al., 2016). Investigation of the Ras signaling pathway has revealed a similar regulatory behavior, in which activation of the proliferative kinase ERK downstream of Ras occurs in discrete bursts, ranging from 20 min to several hours in length, with the duration and frequency of bursts modulated by growth factor concentration, autocrine signals, and cellular density (Aoki et al., 2013). Tracking of single cell responses showed substantial cell-to-cell variation in ERK signaling dynamics, prompting genetically identical sister cells to make different decisions to enter S-phase (Albeck et al., 2013). *In vivo* monitoring of ERK activity reveals similar patterns of pulsatile signaling, indicating that signaling operates in a similar manner under physiological conditions (Hirata et al., 2015). These surprising patterns of activity reveal a new level of complexity in the response to signaling cues, supporting the concept that time-dependent cell-to-cell variation in signaling dynamics contributes to the generation of heterogeneous phenotypes.

### Divergence in gene expression

The functional output of many signaling pathways involved in cancer is the regulated expression of a defined subset of genes. Accordingly, recent studies have focused on correlating the dynamic activity in a signal transduction pathway to the resulting downstream changes in gene expression level (Tay et al., 2010; Lee et al., 2014; Porter et al., 2016; Wilson et al., 2017). At the level of transcription itself, tracking of mRNA abundance reveals that transcription of many genes occurs in the form of “bursts,” in which multiple copies of mRNA are produced, and which are interspersed by dormant periods where no transcripts are made (Suter et al., 2011). The upsurge in mRNA in response to an upstream signal can come from either an increase in the length or frequency of bursts, or an increase in the rate of transcript production during the bursts; interestingly, both scenarios can be observed for the same gene in response to different stimuli (Molina et al., 2013). While a simple model might suggest that the pulses in upstream signals (activity of p53, ERK, NF-kb) correspond to bursts in mRNA production, this does not appear to be strictly the case; for instance, no transcriptional bursting was observed in ERK target genes even when bursts of upstream ERK activity are enforced using optogenetic stimulation (Wilson et al., 2017). Within the context of an ordered tissue, probabilistic gene expression can enhance the dynamic range of regulated gene expression, because the response of multiple neighboring cells can be averaged (Garcia et al., 2013). Theoretical studies also support the idea that cell-to-cell variability can enhance the reliability of signaling at the tissue level (Suderman et al., 2017).

Another potential driver of divergent gene expression between individual cells is the ability of time-dependent processes of mRNA and protein translation to discriminate transient input signals from chronic ones. This effect may be particularly important in Ras/ERK-stimulated gene expression, where ERK is known to control the expression of many of its target genes, such as Fra-1—a transcription factor controlling metastatic behavior—through a “feedforward” regulatory circuit that modulates multiple steps in the expression process (Murphy et al., 2004). For example, a sufficiently long burst of ERK activity may be capable of both stimulating mRNA production (by phosphorylating Elk-1 and other transcription factors) and stabilizing the Fra-1 protein product through phosphorylation once it has eventually been translated. Conversely, a shorter pulse of ERK activity long enough to stimulate the mRNA production step but terminating before translation has been completed would fail to produce phosphorylated (and stable) Fra-1 protein (Murphy et al., 2002). Given that Ras/ERK signaling is often highly dynamic (as discussed above), such temporal filtering may be important in determining the particular gene expression program resulting from a cue that stimulates the Ras pathway. It has also been proposed that expression of dual specificity protein phosphatases (DUSPs), ERK target genes that feed back to dephosphorylate nuclear ERK, may act to bias gene expression toward transient rather than constant activity (Wilson et al., 2017). Moreover, recent studies suggest that there may be yet more diversification in the expression process. Surveys of the mRNAs produced by ERK or p53 activation reveal a diversity of parameter values, such as mRNA half-life, that result in different temporal responsiveness among targets of the same gene (Porter et al., 2016; Uhlitz et al., 2017). Together, these temporally-modulated sources of diversification may effectively allow the same pathway to induce very different gene expression profiles, depending on the duration of pathway activation. Accordingly, predicting single-cell gene responses to stimuli or inhibitors will require mapping each gene's input-output relationship at the single-cell level. However, such data are now in reach, using genome engineering to insert fluorescent reporters at endogenous gene loci and to track expression levels over time in response to defined signaling events (Gillies et al., 2017; Wilson et al., 2017).

Altogether, single-cell studies of signal transduction-mediated gene regulation have revealed many layers of output diversification in response to stimuli. Practically, for our two hypothetical cells, these sources of variation could allow them to exhibit different intracellular responses even in the absence of substantial variation in their microenvironment. This provides yet another possible mechanism for the generation of intratumoral heterogeneity, and more importantly, a mechanism by which cells arising from clonal populations can diverge in their sensitivities to chemotherapeutic agents, as we discuss below.

## From gene expression to drug resistance

After the signaling processes described thus far, our two hypothetical cells are likely to be quite different in their gene expression profile, even though their genomes remain identical in sequence. These expression differences may result in divergence in their threshold for drug tolerance, such that upon exposure to a similar concentration of a cytotoxic drug, one will cell survive while the other succumbs to the treatment (Figure 1, Stage 4). Similarly, the cells may have different capacities for surviving stressful situations that arise physiologically, such as hypoxia within the TME. In this section, we consider how the gene expression changes that have accumulated may result in these divergent survival responses.

### Drug resistance as a function of gene expression

One major factor determining drug resistance is the expression of members the ABC transporter family, which include the multi-drug resistance (MDR) genes. These transporters are capable of exporting various compounds from the cytoplasm, including chemotherapeutic drugs, and their expression thus increases cellular tolerance of cytotoxic therapies. Expression of the ABC transporters is known to be regulated by Wnt signaling, multiple microRNAs, the transcription factors Nrf2 and Runx3, and the histone methyltransferase EZH2 (Chen et al., 2016), making it possible for microenvironmental signals to modulate transporter levels. There are at least three ABC transporter genes involved in cancer drug resistance—P-glyocoprotein/MDR1 (ABCB1), MRP2 (ABCC2), and BCRP (ABCG2)—which have distinct, but overlapping, spectra of substrates. Thus, the threshold of drug tolerance mediated by MDR expression can be expected to vary as a function of signals received from the microenvironment, but is difficult to predict for any individual cell due to its multi-factorial nature.

Also important in determining cellular drug sensitivity are regulators of apoptosis, including the Bcl-2 family of proteins, because many chemotherapeutic agents induce cell death through apoptosis. There are at least 17 genes in the Bcl-2 family in humans, with both pro- and anti-apoptotic roles, and the overall threshold for the induction of apoptosis is set by the aggregate levels of these opposing proteins (Certo et al., 2006). The expression of many Bcl-2 family members is under control of signaling pathways that lie downstream of TME signals (Holohan et al., 2013). Similarly, expression levels of the many components of the DNA damage repair machinery can determine the cellular tolerance for DNA-damaging therapies (Bouwman and Jonkers, 2012). Expression profiles can also affect drug sensitivity indirectly; for example, expression levels of cyclins and CDK inhibitors modulate the rate of cell cycle progression, which can in turn determine the sensitivity to chemotherapeutics, such as microtubule stabilizers, that target cells at specific stages of the cell cycle. Thus, the overall ability to tolerate drug exposure is determined by the composite expression levels of dozens of proteins, some of which have specificity for certain drugs or drug classes, and others which control cell death responses more generally. Drug resistance therefore behaves as a complex trait of individual cells.

### Connecting gene expression profiles to cell states

With ~20,000 genes in the human genome, there are an extremely large number of possible expression profiles for each cell, even if it is assumed that each gene has only two expression states (“on” or “off”) and that many genes are coordinated in their expression status. Of course, many of these states are in reality either unstable or unreachable due to conflicting regulation, such as the simultaneous expression of two transcription factors that each inhibit the other's transcription (Brock et al., 2009). Conversely, certain states are self-reinforcing due to positive feedback regulation, leading to the concept that there are “attractors”—stable regions within the overall space of gene expression profiles where cells tend to cluster (Huang et al., 2005). Considering our two cells that began with the same expression profile, a key question is how far these two cells may diverge in their overall expression status—potentially crossing from one attractor state to another—and whether this divergence will affect their metastatic and chemoresistance properties.

Functional studies support the relevance of attractor states for tumor cells, and suggest that tumor cells may transition between discrete expression profiles correlating with drug resistance (Ponti et al., 2005; Chiba et al., 2006). Well known examples of such states and transitions include the EMT and CSC states identified within some cancer types, prompting the model of a dynamic equilibrium of cell states underlying tumor heterogeneity (Gupta et al., 2011). However, precisely defining the expression profiles corresponding to these states remains challenging and will likely require single-cell resolution. For example, if high expression levels of genes A and B together create a drug resistant state, single-cell methods will be needed to detect such cells because population-based methods cannot distinguish whether A and B are co-expressed within the same cells or separately in two different subpopulations of cells. While genome-wide expression profiling for large populations of cells has been possible for more than 15 years, practical methods for single-cell profiling have only recently become widely available and remain limited by the inherent technical challenges in accurately sampling the ~1 pg of mRNA present in each cell. Moreover, single-cell profiling provides only a static snapshot of the expression profile at the time of the assay and provides little information on dynamic transitions, making it difficult to link to functional properties such as drug resistance. Nonetheless, as they mature, single-cell profiling technologies promise to provide molecular clarity in mapping the cell states accessible to tumor cells. Of particular interest are methodologies capable of tracking dynamic cellular behavior over time and correlating this behavior with the genome-wide expression profile within the same cell (Lane et al., 2017).

A key question is therefore whether the expression profile of tumor cells can be used to accurately predict drug responsiveness. Such tests, based on population-level measurements of mRNA abundance, are clinically available and have prognostic value (van 't Veer et al., 2002; Paik et al., 2004; Drukker et al., 2014; Shah et al., 2017). However, single-cell resolution of gene expression profiles are now feasible and could in principle more accurately predict clinical of interest (Anjanappa et al., 2017), since resistant and metastatic cells may be present as minor subpopulations that are obscured by the bulk of the tumor and any contaminating non-tumor cells. A remaining challenge is to ascertain whether there are discrete cell states identifiable by expression signatures that are broadly predictive of tumor cell characteristics. In this regard, it will be critical to determine whether attractor states corresponding to drug resistance behavior do in fact exist and can be detected by their expression profile. An alternative possibility is that the many layers of variation in gene expression, as discussed above, create a continuous landscape of expression states rather than discrete cell types; this could substantially complicate the analysis of tumor subpopulations.

### Maintenance of drug-resistant states over time

States of drug tolerance may persist for times ranging from hours to weeks. Some drug-resistant states have been attributed to epigenetic mechanisms (Sharma et al., 2010). Typically, the term epigenetics is used to refer to chromatin modifications, including DNA methylation and histone acetylation or phosphorylation that can modulate gene expression patterns and which persist across multiple cell generations (Easwaran et al., 2014). These covalent modifications can play a role in resistance to chemotherapeutics are often highly stable, allowing a particular gene expression profile to persist for weeks or longer. However, they may be reversed by inhibitors of chromatin-modifying enzymes, accelerating the loss of the resistant phenotype (Sharma et al., 2010).

Considered more broadly, the concept of epigenetics includes any heritable cellular trait controlled by factors other than nucleic acid sequence. For example, protein expression levels can vary between genetically identical cells, and these differences can be preserved through cell division, making related cells more likely to contain similar protein expression levels (Sigal et al., 2006). Life-or-death differences in cell fate can result, as fluctuations in the levels of Bcl-2 family and other proteins can determine sensitivity to apoptosis inducers such as TRAIL or chemotherapeutics (Spencer et al., 2009). Because of the relatively short time needed for protein turnover to reshuffle expression levels, such states tend to persist for shorter periods of time, typically from hours to a few days (Flusberg et al., 2013; Flusberg and Sorger, 2015). In such cases, the cells surviving a cytotoxic treatment repopulate the original distribution, enabling a similar fraction of cells to be killed by a second round of the same treatment. Quantifying the time needed for the redistribution of resistance properties can thus be useful for determining the optimal frequency of cytotoxic treatments.

## Looking forward

We have traced here the flow of information from heterogeneous extracellular signals originating in the microenvironment, through variance-prone signaling networks, to regulate cell fate at the level of gene expression, emphasizing that this process tends to diversify rather than constrict cellular responses to a narrow range (Figure 1, Stages 1–4). In the context of a normal tissue, such diversity may be important for maintaining proper tissue function, for example by maintaining subpopulations of cells prepared to respond to a broad range of stimuli or stressors, or by increasing the dynamic range of the mean response (Suderman et al., 2017). In the context of a tumor, where signals are spatially and temporally heterogeneous, the same properties likely contribute to tumor cell resilience by creating diverse subpopulations with selective survival advantages (relative to normal cells) as they disseminate to foreign environments and evade therapy. The key question that now arises is how these advances in understanding the molecular diversity of cancer cells can be translated into more effective therapies (Brock et al., 2009). We consider here how single-cell technologies may impact the diagnosis and the development of new compounds or treatment regimens for cancer.

### Correlating prognosis and treatment efficacy with single-cell measurements

Ideally, transient therapy-resistant subpopulations (or subpopulations with the potential to become resistant) could be detected, and appropriate treatment strategies chosen depending on the distribution of single-cell profiles within each patient's tumor. Yet, while the technology for measuring such heterogeneity is now available, in the form of methods for single-cell sequencing of genetic and transcriptional profiles, significant obstacles remain before this information can be fully deployed in predicting patient responses to therapy.

First, it remains to be determined whether accurate detection of non-genetic heterogeneity and characterization of subpopulations within a tumor is practically feasible within a clinical setting. Single-cell sequencing of genomic DNA from nuclei in frozen tumor sections has established technical feasibility of single-cell isolation and sequencing and has demonstrated that sampling of 50–100 cells was sufficient to capture all of the predominant clones with a high level of confidence (Gao et al., 2016). However, the greater variability in transcriptional profiles may require the sequencing of a substantially larger number of cells to detect rare subpopulations. This number will depend on both the complexity of the population as well as the size and spatial heterogeneity of the tumor. Identifying clear predictive trends in such data also faces a significant statistical barrier, because in increasing the complexity of tumor classification, it becomes difficult to include sufficient numbers of patients to power statistical tests. Finally, it will be necessary to integrate models of variability in the genome and transcriptome to understand clonal evolution of tumors over time. Modeling of the clonal diversity within tumors has revealed a complex interplay by which certain clones play a supportive role for other cells within the tumor through secreted factors, and elimination of these clones can broadly reduce tumor cell viability (Marusyk et al., 2014). However, such changes can also enhance the viability of other clones (Waclaw et al., 2015), making the overall outcome difficult to predict with our current understanding.

Regardless of the specific technologies used, much still depends on better models linking transcriptional profiles to cellular phenotypes. This remains a daunting challenge; even at the bulk tumor level, predicting treatment response from genetic or transcriptomic profiling remains difficult for the majority of cancers (Niepel et al., 2017), despite a small number of high-profile examples in which a driver mutation predicts drug responsiveness (e.g., HER2 amplification in breast cancer or B-Raf mutation in melanoma). Interestingly, measuring signaling responses provides a more effective means than genetic markers for predicting drug sensitivity in cell culture models (Niepel et al., 2013), suggesting that single-cell resolution could improve accuracy by revealing the constituent subclones and their sensitivities. Further advances will require moving from reductionist cell culture systems to more realistic models that incorporate the effects of the microenvironment.

### Therapeutic strategies to counteract cellular heterogeneity

The concepts presented here imply that therapeutic strategies should target not simply the central tendencies and static genetic complement of a tumor, but also the many subpopulations of transient cell states co-existing within the tumor and their potential to change their signaling and transcriptional profile in response to drugs (often termed an adaptive response). Early attempts to address this complexity searched for drugs that selectively target cancer cells with high potential to reinitiate tumors (Gupta et al., 2009). Other studies, recognizing that drugs shift the signaling behavior of tumor cells to induce resistant cellular phenotypes, have identified pathways involved in this adaptation and demonstrated the effectiveness of simultaneous inhibition of these pathways (Tandon et al., 2011; Rexer et al., 2014). These advances notwithstanding, there remains further potential to use information on cellular heterogeneity to improve therapeutic responses.

A clear demonstration of the role of variability in drug sensitivity is the long-standing observation that multiple rounds of chemotherapy are typically more effective than single treatments. If genetic variability alone were the cause of drug resistance, the surviving cells would all be genetically resistant and no benefit would be achieved from additional rounds. By understanding the mechanisms and kinetics of transitions between sensitive and resistant states, the timing of drug treatments can be better tailored to maximize the number of cells responding (Flusberg and Sorger, 2013). Alternatively, therapeutic approaches that reduce the intracellular heterogeneity of gene expression prior to treatment with a cytotoxic drug could improve the efficacy of tumor cell killing. A number of compounds that alter chromatin modification, such as bromodomain inhibitors, may be useful in this regard by preventing cells from entering resistant transcriptional states (Sharma et al., 2010). Conversely, antibodies blocking the effects of extracellular components may be used to limit the impact of the microenvironment in generating intratumoral heterogeneity. While the first generation of such molecules, such as inhibitors of VEGF or matrix metalloproteases have limited efficacy, the potential remains for multi-pronged interventions to normalize the TME. One recent study has provided an exciting example of how crosstalk between tumor cells and CAFs orchestrates the divergence of basal and ER-positive subtypes of breast cancer and can be interrupted by inhibitors to revert tumor cells to a more easily treated subtype (Roswall et al., 2018). In addition, immune system-based approaches promise to provide a new arsenal of tools that could be less dependent on the native microenvironmental heterogeneity.

### Further advances in understanding the fundamental mechanisms of variation

A limitation of many of the studies discussed here is that they each primarily investigate a single pathway, and provide little insight into how signaling networks function under physiological conditions when multiple pathways converge. Significant technical limitations remain for understanding how multiple microenvironmental signals are integrated by individual tumor cells, in a dynamic way. At the imaging level, the maximum number of pathways that can be simultaneously interrogated in a live single-cell remains ~4 (Regot et al., 2014). At the analysis level, methods for signal engineering have yet to be adapted for use on the relatively sparse and noisy data from live-cell experiments. Single-cell mRNA sequencing has provided a wealth of data on where cells cluster within the many-dimensional space of possible gene expression patterns. Application of this technology has already vastly expanded our knowledge of the phenotypic and stroma cell states present in the TME. However, these data are snapshots and do not reveal how frequently transitions are made between cell states. Nonetheless, further studies using high-content imaging and single cell genomic approaches will allow interrogation of individual cells within a population to understand how signaling dynamics are integrated and determine gene expression programs. Combining these modalities with physiologically relevant 3-dimensional, and multicellular, culture models will allow us to measure tumor–stroma signal cross-talk with new precision. Importantly, further development of computational methods and models will be essential to interpret these complex experiments. Combining these approaches will more accurately determine the relative contributions of extrinsic and intrinsic factors to cell fate determination. In doing so we will gain valuable insight into how these factors contribute the plasticity of tumors and ultimately how to control them for therapeutic benefit.

## Author contributions

All authors listed have made a substantial, direct and intellectual contribution to the work, and approved it for publication.

### Conflict of interest statement

The authors declare that the research was conducted in the absence of any commercial or financial relationships that could be construed as a potential conflict of interest. The reviewer MRS declared a shared affiliation, with no collaboration, with one of the authors, AED, to the handling Editor.
